# Un volumineux ostéome orbitaire: à propos d'un cas

**DOI:** 10.11604/pamj.2014.18.10.3603

**Published:** 2014-05-02

**Authors:** Kamal Loutfi Nuiakh, Hicham Tahri

**Affiliations:** 1 Service d'Ophtalmologie, CHU Hassan II, Fès, Maroc

**Keywords:** Ostéome, exophtalmie, orbite, osteoma, exophthalmos, eye-socket

## Image en medicine

Patient âgé de 56 ans, sans antécédent pathologique particulier, présentait depuis 5 ans une exophtalmie droite (A), progressive, non axile en bas et en dehors, non réductible et indolore. L'examen ophtalmologique trouvait une perception lumineuse négative droite avec une atrophie optique. La tomodensitométrie orbitaire montrait un processus fronto-éthmoïdal de densité osseuse avec une extension intra-orbitaire fortement évocateur d'un ostéome (B, C). Une abstention thérapeutique avec surveillance clinique était préconisée vu la nature bénigne de la tumeur et la perte fonctionnelle de l'oeil. L'ostéome orbitaire est une tumeur bénigne à croissance lente. Le potentiel agressif est lié essentiellement aux complications orbitaires et parfois endocrâniennes. Cette observation nous a paru intéressante à rapporter du fait de la rareté des ostéomes éthmoïdaux à évolution orbitaire.

**Figure 1:**
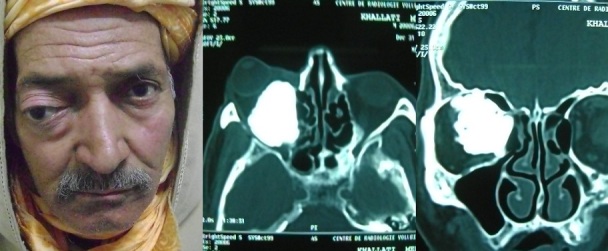
A) exophtalmie droite, non axile en bas et en dehors; B) coupe axiale à la tomodensitométrie montrant un processus fronto-éthmoïdal de densité osseuse avec une extension intra-orbitaire; C) coupe coronale à la tomodensitométrie montrant le processus fronto-éthmoïdal de densité osseuse

